# Leveraging Single-Cell Populations to Uncover the Genetic Basis of Complex Traits

**DOI:** 10.1146/annurev-genet-022123-110824

**Published:** 2023-08-10

**Authors:** Mark A.A. Minow, Alexandre P. Marand, Robert J. Schmitz

**Affiliations:** Department of Genetics, University of Georgia, Athens, Georgia, USA

**Keywords:** single-cell genomics, quantitative genetics, gene expression, chromatin accessibility, mutants, population genetics

## Abstract

The ease and throughput of single-cell genomics have steadily improved, and its current trajectory suggests that surveying single-cell populations will become routine. We discuss the merger of quantitative genetics with single-cell genomics and emphasize how this synergizes with advantages intrinsic to plants. Single-cell population genomics provides increased detection resolution when mapping variants that control molecular traits, including gene expression or chromatin accessibility. Additionally, single-cell population genomics reveals the cell types in which variants act and, when combined with organism-level phenotype measurements, unveils which cellular contexts impact higher-order traits. Emerging technologies, notably multiomics, can facilitate the measurement of both genetic changes and genomic traits in single cells, enabling single-cell genetic experiments. The implementation of single-cell genetics will advance the investigation of the genetic architecture of complex molecular traits and provide new experimental paradigms to study eukaryotic genetics.

## INTRODUCTION

Cellular differentiation into discrete biological roles underpins the success of multicellular life, allowing the organism to have more functions than the sum of its cellular constituents. However, this complex cellular milieu complicates any biological measurement from bulk tissues, as abundant cell-type signals mask those from less prevalent cells. Single-cell genomics provides the power to deconvolute the biological signals from bulk tissues. Single-cell genomics, although only in its nascent stage, is set to trigger a revolution in biological research analogous to high-throughput sequencing in the 2000s. Beyond allowing researchers to better study specific cellular contexts, the ability to measure individual cells significantly increases the resolution and power of many preexisting techniques, including quantitative genetic studies.

Most heritable phenotypes are influenced by alleles at many unlinked loci. These complex, quantitative, or polygenic traits are often of utmost biological importance, including phenotypes such as disease susceptibility, developmental processes, physiological age, animal productivity, and crop yield (40, 72). Discovering the genetic drivers of quantitative traits involves measuring both the phenotype and genotype of a genetically diverse population. Then, through statistical associations, individual quantitative trait loci (QTL) can be implicated in controlling the measured phenotype. These population studies are effective at finding large-effect QTL. However, since many loci control quantitative traits, dissecting all loci that underpin their heritability remains a challenge, leading to missing heritability that even studies with thousands of individuals lack the power to find (44, 67, 79, 80, 152, 162). Although presently unrealized, single-cell genomics may empower population genetics to unveil more of the genetic architecture behind quantitative traits.

Plant research is unique in that its applied product is the plants themselves, with much applied research aimed at breeding progressively better plant genotypes. Artificial selection on plants, by ancient and modern humans alike, has shaped plant products to meet human needs. Prehistoric hunter-gatherers unwittingly altered plant genomes when domesticating crop species, precipitating the agricultural revolution and the birth of civilization. Likewise, modern breeding applied quantitative genetics and genomics to release cutting-edge crop varieties, which are largely responsible for the increases in agronomic yield since the Green Revolution. The intense interest in plant quantitative genetics, along with several other aspects of plant biology, has repeatedly translated value to the broader research community, with plant systems pioneering techniques including blocking and split plot designs (35, 106). These techniques account for the effects of impractical-to-control variables (e.g., the effect of hospitals on health outcomes or fields on plant growth) by distributing their effects evenly between individuals in a study. As has been the case historically, we believe that plant biology has unique advantages that will work in tandem with the single-cell genomics revolution to spearhead further human understanding of heritable quantitative traits.

Here, we frame the results of pioneering mammalian single-cell population studies against the backdrop of classical plant genetic questions, highlighting the potential of applying single-cell genomics to plant populations. We also outline how established plant genetic phenomena and resources can integrate with single-cell tools to usher in an era of single-cell genetics. We anticipate that further application of single-cell approaches will reveal much about quantitative traits and translate to a better understanding of the regulatory relationships between loci in eukaryotic genomes.

## SINGLE-CELL SEQUENCING FROM PAST TO PRESENT

The first single-cell transcriptome was published in 2009 from a mechanically isolated mouse blastomere (137). Current single-cell sequencing techniques use combinatorial indexing, microfluidics, or both to distinctly isolate and label individual cells or nuclei. Combinatorial indexing, which uses sequential indexing and mixing steps to give each cell a unique identifier, was originally used to measure 34 cellular transcriptomes (52). Microfluidic separation and tagging of cells increased individual measurements considerably, with early reports sequencing ~45,000 cells (78). The ease of single-cell sequencing has increased such that both microfluidic separation and combinatorial indexing “omic” techniques routinely measure ~10^4^ cells per sample, and this is poised to increase tenfold in the immediate future (23, 65).

Single-cell methods have evolved to measure genome sequence, cell surface proteins, small RNAs, DNA methylation, histone modifications, chromatin accessibility, chromosomal conformation, and proteomes (20, 107, 130). Techniques have also been developed that measure two or more molecular traits from the exact same cell. These multiomic measurements are usually dependent on transforming biological signals into DNA-level information, which can be deconvoluted via sequencing. Some of these signals, such as DNA polymorphism detection, are inherent to sequencing techniques, whereas other multiomic measurements are molecularly engineered into the experiment, such as DNA tagging antibodies or sequencing CRISPR-CAS9 deletion cassettes to determine targets post hoc (26, 111, 130). Sequential read indexing steps for distinct genomic measurements can produce multiomic data, with multiomic single-cell measurement of RNA expression and chromatin accessibility becoming more common.

Cell functional divergence involves different uses of the genome, and these differences can occur via the altered usage of *cis*-regulatory elements (CREs) located within accessible chromatin regions (ACRs). Chromatin accessibility is measured through the biased restriction of enzymatic activity to nucleosome-depleted chromatin that houses the DNA regulatory motifs regulating gene expression. Chromatin accessibility is routinely determined via assay for transposase-accessible chromatin sequencing (ATAC-seq) (11). ATAC-seq utilizes Tn5, a hyperactive transposase, to insert sequencing adapters directly into accessible chromatin and is used for single-cell measurements (scATAC-seq). Since gene accessibility and RNA expression are only loosely correlated [Spearman’s correlation coefficient 0.54–0.58 (81), −0.07–0.77 (22); Pearson’s correlation coefficient 0.22–0.26 (141)], single-cell RNA sequencing (scRNA-seq) complements scATAC-seq, with the integration of both data types providing a more comprehensive depiction of cellular states. Currently, both scATAC-seq and scRNA-seq only measure a small fraction of the transcripts or ACRs in a cell (resulting in high data sparsity), which, when combined with the large number of measurements, complicates the corresponding data analytics (reviewed in 4, 121). After analysis, both techniques place related cells into clusters, which can be analyzed independently. This cell type–level clustering prevents abundant cell-type signals from drowning out signals originating in rarer cells. In this way, scATAC-seq unveiled which genes and ACRs exhibit cell-type enrichment, as well as the prevalence of heterogeneity and subtypes within individual cell types (82). Even in animal models, which, unlike plants, possess pure cell culture lines, scATAC-seq provided a more representative measure of the in vivo cell-type reality (107). Likewise, scRNA-seq has deconvoluted transcriptomes, facilitating the discovery of novel cell-type functions, including the previously unknown ultraviolet (UV) protectant role of *Arabidopsis* palisade mesophyll cells (108).

The cell wall has been a barrier to the migration of techniques from animal systems to plant models. However, single-cell techniques, such as scATAC-seq and scRNA-seq, can be performed on isolated nuclei instead of cells (34, 81). Analyzing nuclei facilitates high-throughput genomic approaches much like in mammalian systems. Additionally, since single-nucleus RNA sequencing (snRNA-seq) detects polyadenylated transcripts before their nuclear export, it removes the measurement of nonautonomous (cell-to-cell mobile) messenger RNA (mRNA), a common plant phenomenon with currently unknown ramifications in single-cell analysis (58). For this reason, snRNA-seq has the potential to be a more accurate measure of mRNA transcription in each cell type. However, measuring nuclei ablates all cytoplasmic information, precluding the observation of signals such as cell proteomes (20). Although most organellar genes are nuclear encoded, assaying nuclei strips each cellularized measurement from its complementary plastids and mitochondria, partially obscuring organelle roles in planta.

Removing walls from plant cells creates protoplasts and enables cell separation techniques analogous to those used with animal cells. Although protoplast-based methods have produced whole-cell transcriptomes (68, 73, 92, 108, 113, 115, 119, 158, 166), we believe the high-throughput nature of nuclei-based single-cell measurements provides a powerful and adaptable tool that will address many outstanding questions in the field. In particular, multiomic measurement of chromatin accessibility and RNA expression in high numbers of nuclei will advance our ability to study transcriptional regulation. Concurrent multiomic approaches result in somewhat lower throughput, as cell-level quality control is stricter when applied to two modalities instead of one. However, similar to the general trend in single-cell approaches, further technical developments will likely lead to improvements in scalability. These multiomic measurements can combine with unique aspects of plant biology to expose how genomes manifest complex phenotypes.

## ASPECTS OF PLANT BIOLOGY THAT SYNERGIZE WITH SINGLE-CELL TECHNOLOGIES

**Figure 1:**
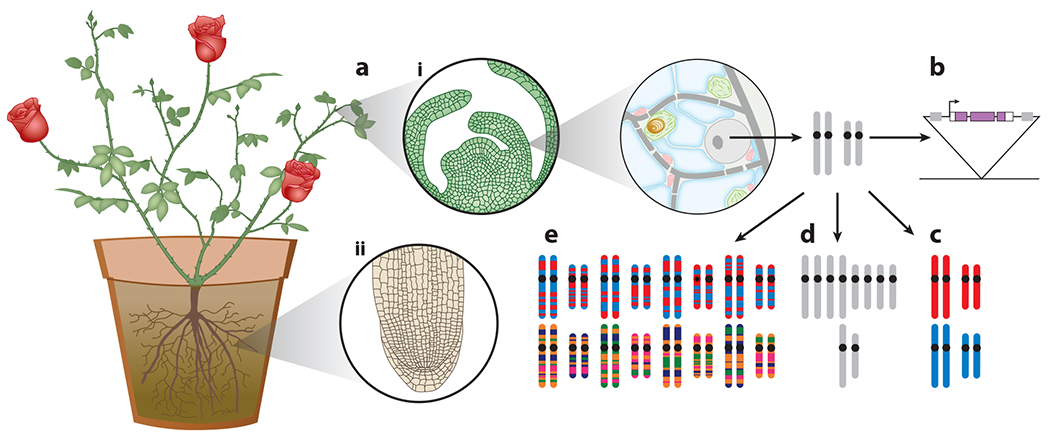
Plant biology provides unique advantages for single-cell analysis. (*a*) Plants develop organs continuously from shoot (*i*) and root (*ii*) meristems, which undergo predictable cellular divisions to produce organs with consistent cellular organizations. This meristematic growth greatly simplifies the sampling of single cells along a developmental gradient. (*b*) Stable transgenesis, a powerful tool to support single-cell techniques and confirm discoveries, is routine in many plant models and expanding in crops and nonmodel species. (*c*) Many plants, notably important crops, tolerate inbreeding to whole-genome homozygosity, aiding any single-cell genetic or genomic inquiries and allowing genotypic replication across treatments or environments. (*d*) Plants tolerate increases and decreases in ploidy, allowing for the interrogation of how different cell types respond to genome-wide gene dosage changes while easing certain single-cell analyses. (*e*) Through controlled crossing and inbreeding, many plant populations have been designed to map and study the genetic variants that underpin trait heritability. Using these resources, single-cell population genomics will provide better resolution to find molecular trait–associated variants while discerning which cell types they affect.

Aspects of plant biology leave it uniquely positioned to address basic genetic questions through single-cell approaches. Plant organs consist of specialized cell types with cellular arrangements semiconserved between species. Unlike mammals, plants undergo continuous growth and development (Figure 1a), with new organs arising from meristematic stem-cell niches throughout their life cycle (120, 125, 129). Within a species, organs are produced by repeating cell-type patterns that are similar between older and younger organs. This continual and consistent organogenesis means that cells exist in progressive stages of maturation at one time point, simplifying the sampling of developmental gradients to study cellular differentiation. Single-cell sampling of these developmental gradients in roots (119, 166) and other organs (81, 115) has rediscovered known master regulators and uncovered the spatiotemporal context in which unstudied transcription factors act (119).

Plants, being sessile, cannot move to escape stressful environments and instead endure them. To withstand stress, plants have evolved to modify their growth and development in response to their environment. This developmental plasticity means one genotype can produce different morphologies (99) and biochemistries in distinct environments, with the extent of plasticity varying between and within species (133). Development and cell function rely upon changes to chromatin accessibility, altering CRE use, and tuning gene expression as needed (99). However, the chromatin changes that tune plastic development or biochemistry remain poorly understood, although some progress has been made mapping loci that influence traits such as organ morphologies (64), and plastic changes to ACR peak shape have been observed (71). Single-cell measurements of mRNA and chromatin within plastic genotypes in different environments will reveal more about the molecular mechanisms and cell types that drive environmental adaptation. We expect that some cell types have oversized roles in specific stress responses (e.g., the stomata, epidermis, and vasculature in response to drought) and that the associated genomic changes in these cell types will be revealed via scRNA- or scATAC-seq. Uncovering the mechanisms behind plant plasticity could ease the selection for beneficial plastic responses or for genetic manipulation to induce the genetic assimilation of beneficial traits.

Transgenesis is a great ancillary tool to single-cell studies, helping to both label cell populations through cell-specific reporters and confirm or elaborate upon any findings. The ease of stable transgenesis in *Arabidopsis* (18) is a considerable asset to investigate the mechanistic roles of interesting genes or CREs highlighted by single-cell genomics. Beyond the ability to create novel transgenics to address hypotheses, *Arabidopsis* has established cell-type reporters that have aided cell-type discovery from single-cell data (108, 168). Unlike *Arabidopsis*, most crops and nonmodel plants rely upon tissue culture–based transformation, making them more recalcitrant to transgenesis. However, methods have been pioneered in maize that use transient expression of growth regulators to generate transgenics via somatic embryogenesis (16). Importantly, the extension of these somatic embryogenesis–based methods to more species will ease the use of transgenic techniques (Figure 1b) to verify and expand upon single-cell genomic findings.

Plant genomes tolerate many genetic manipulations better than metazoans, including changes in ploidy (Figure 1c). Organogenesis in flowering plants incorporates endoreduplication (24), and the generation of triploid endosperm during reproduction is required for viable embryogenesis. Moreover, angiosperm lineages have undergone whole-genome duplications, or polyploidizations, during their evolution (144). These historical polyploidizations are thought to have provided diversity and functional redundancy during stressful geological eras, fueling plant divergence in form and function (144). Single-cell study of these systems will reveal how individual cell types respond to ploidy changes, providing insights into how genomes adapt to these differing gene doses. Plants also endure reductions in ploidy, with all plants consisting of a haploid gametophytic stage, and many angiosperm (sporophytic) haploids develop to sexual maturity (31). Due to the breeding benefits of doubled haploids, haploid induction techniques have been created in a wide range of crops (56, 59, 148, 160) and models such as *Arabidopsis* (110). The reduced genetic redundancy in haploids makes all alleles fully penetrant and reduces the sequencing depth needed to cover the genome, a great asset for single-cell approaches. These established ploidy manipulation systems are an asset in designing plant single-cell studies, notably those investigating how ploidy changes alter cellular development.

Many plants, notably crops, can exist in a highly inbred state, leaving each locus effectively homozygous (Figure 1d). These inbred genotypes often have complete and annotated genomes, for example, *Arabidopsis* (1, 57, 165), maize (51, 55, 118, 126), tomato (49, 131, 135), rice (109) and soy (70, 75). Having only one allele variant present greatly simplifies allelic functional characterization. Moreover, genomic homozygosity allows for genotypic replication across different conditions, which is impossible in species intolerant of inbreeding. The inbreeding tolerance of many plants, along with controlled crosses, facilitates the generation of powerful population-level tools (Figure 1e). Beyond classical biparental recombinant inbred lines (RILs) (5), other population designs, including nested association mapping (NAM) (37, 63, 124, 164) and multiparent advanced generation intercross (MAGIC) populations (63, 77), have been created that captured more genetic diversity and improved trait-mapping resolutions. The maize NAM population is accompanied by complete reference genomes (51), facilitating the functional dissection of allelic variants typically missed by short-read sequencing and dependence on a single reference assembly. In contrast to inbreeding tolerance, some plants withstand wide hybridization events, with certain interspecific crosses producing viable offspring and with cross-species introgression driving plant trait evolution (140). Many crops have extant wild progenitors or relatives that retain sexual compatibility with their domesticated counterparts. This has been exploited to create recombinant populations segregating domesticated and wild alleles (3, 6, 15). These populations provide a unique opportunity to study genome domestication, in addition to identifying wild alleles for crop improvement. Furthermore, the many polymorphisms between domesticated and wild transcripts provide a great opportunity to track allele-specific expression, simplifying the study of how domesticated alleles have changed. The combination of these established populations with single-cell population genomics will be helpful in learning more about both domestication and the genetic control of phenotypes.

## QUANTITATIVE GENETICS HAS SHAPED OUR UNDERSTANDING OF COMPLEX TRAIT INHERITANCE

**Figure 2:**
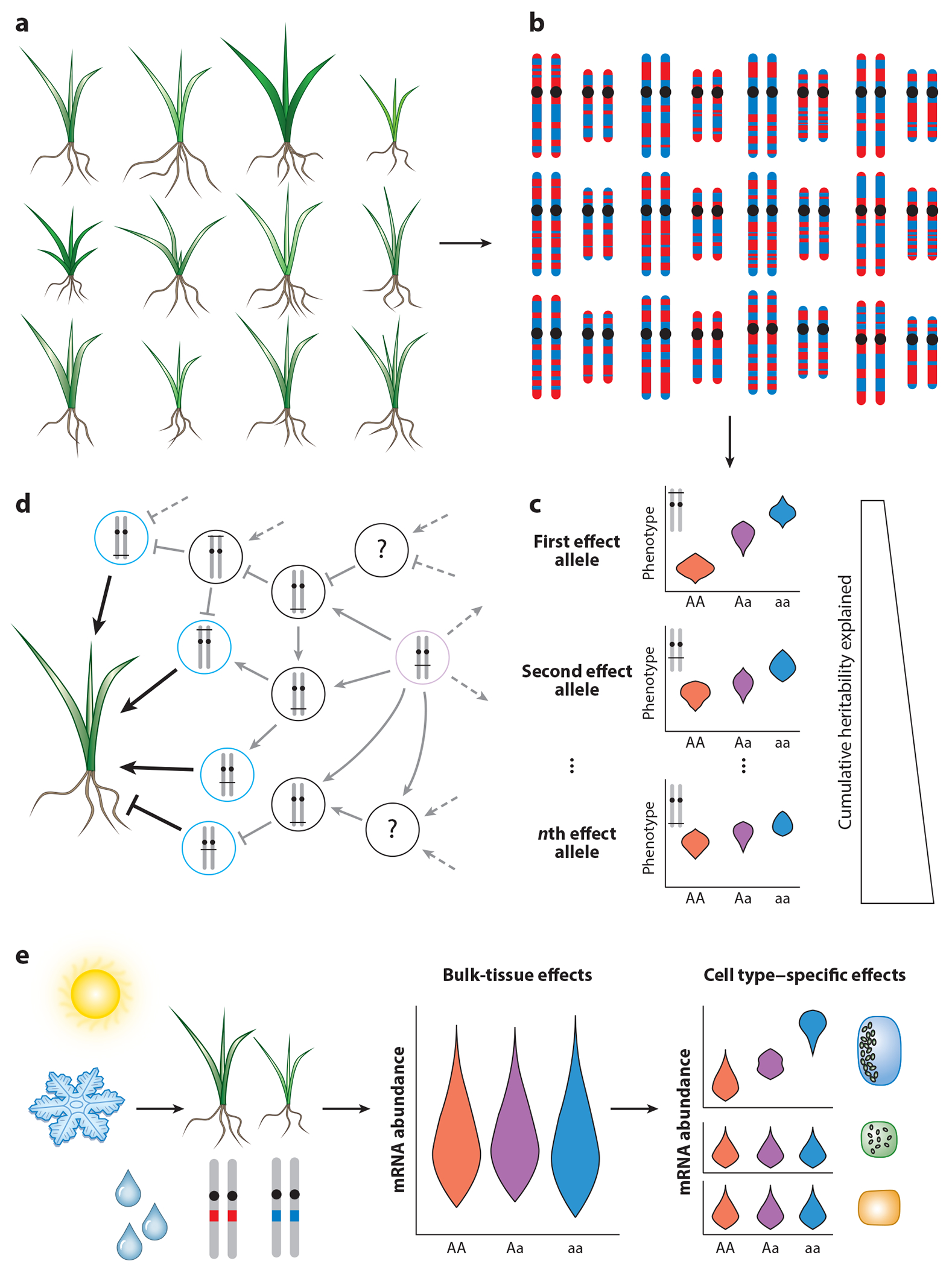
Integrating measurements of genetically diverse populations with single-cell techniques. (*a*) Historically, population genetics required a population of individuals with variation in heritable phenotypes. (*b*) The individual genotypes in this population are determined and paired with phenotypic measurements. (*c*) Paired genotypes and phenotypes enable the trait comparison between the subpopulations with various complements of all alleles. Different alleles linked to loci that variably control a trait should have discernable effect sizes on phenotypes. As more regions controlling phenotypes are discovered, more of the heritability underpinning a trait can be explained. (*d*) Study of trait heritability revealed that complex traits are controlled by a few loci with large phenotype effects and many loci that have infinitesimally small impacts, leading to the omnigenic model of inheritance. In this model, variants in a few core genes (*blue circles*) directly control traits, having a large effect on phenotype. These core genes are in turn regulated by peripheral genes (*black circles*), including peripheral master regulators (*purple circle*). As these peripheral regulators descend into the organismal gene regulatory network, they have a diminishing effect on phenotypes and become more difficult to discover. (*e*) Measuring molecular traits in single cells from genetically diverse individuals reveals variants that have impacts in rare cell types previously obscured in bulk assays. Combined with the ability to ascertain which cell types variants impact, the increased discovery power of single-cell assays will provide more insights into the genetic architecture driving phenomena such as plant environmental responses and development. Abbreviation: mRNA, messenger RNA.

Continuous phenotypes, such as disease susceptibility, height, crop yield, and stress tolerance, are influenced by both environmental and hereditary factors, with heritability referring to the proportion of the population phenotypic variance explained by genetics. The polygenic or quantitative genetics that underpins the heritability of continuous phenotypes is complex, encompassing the contributions of many unlinked genes. To uncover the loci controlling continuous traits, a population with genetic and trait diversity must be selected and the phenotypes and genotypes of individuals within it measured (Figure 1c). If a variant affects a quantitative trait, there should be a discernible phenotypic difference between individuals with or without the variant of interest (Figure 2c). Early quantitative genetics used crosses between two phenotypically different parents to generate recombinant progeny, segregating both known genetic markers and unknown genes controlling a trait. Through tracing recombination and quantifying individual phenotypes, it became possible to know the number of QTL in a population and their genetic locations (138). Molecular genetic markers provided better and more ubiquitous coverage of the genome, facilitating the mapping of the first disease-causing gene through similarly tracking recombination (45). Array hybridization technology expanded the ability to measure polymorphisms, often single-nucleotide polymorphisms (SNPs), while also facilitating early transcriptomics. These arrays lead to the first expression QTL (eQTL) studies, which used gene expression as a quantitative trait and mapped the genetic variants that influence individual gene expression genome-wide (8, 116). eQTL analysis can uncover both proximal (*cis*) and distal (*trans*) genetic variants affecting gene expression (94). *Trans* effects tend to have smaller effect sizes than *cis* equivalents, and even concurrent studies with >10^4^ individuals can lack the power to exhaustively find all *trans* regulators (94, 147).

Although powerful, tracking recombination between biparental alleles is often impractical in real-world populations. By comparing phenotypes between individuals with a variant of interest to the rest of a study population, genome-wide association (GWA) can uncover loci influencing polygenic traits in populations segregating for many divergent haplotypes, taking advantage of historical recombination to partition large haplotypes. Pioneered in the 2000s (61, 98), GWA has since been applied to many populations where it has unveiled molecular mechanisms controlling complex traits in humans, plants, and animals (64, 128, 136). However, GWA is complicated by statistical type I errors due to multiple testing, the difficulty in tying significant loci to causal DNA changes, and the confounding effects of shared ancestry (population structure) causing the coinheritance of noneffect alleles with causal variants (136).

Quantitative genetics has advanced our understanding of the functional genome and found loci with large trait effects, providing tangible benefits to disease research (136) and enabling genomic selection during plant and animal breeding (46, 47). Quantitative genetics has also revealed the importance of rare, large-effect alleles in standing variation (17, 39, 62). The large contribution of rare alleles to phenotype has breeding relevance, as a too-stringent selection cutoff may prevent the advancement of important variants. Paradoxically, these ultrarare alleles are recalcitrant to quantitative genetic study due to low allele frequency (low *n*) translating to poor statistical power. This problem can be improved by pooling different variants (105) in similar features (e.g., treating variants in the same exon as equivalent alleles) or generating biparental populations to increase allele frequency.

Even when examining traits with high heritability, quantitative genetics often only finds a small fraction of the loci controlling a trait (76, 85). Illustrating this missing heritability, pioneer quantitative genetic studies of human height explained ~5% of human height variability, a trait that is 80% heritable (44, 67, 152). Plant models, owing to their high genetic diversity, controlled crosses, and propensity for genotypic replication, can provide an exception to this rule. For example, a study of a maize diversity panel explained 89% of time-to-flowering heritability (10) and a high proportion of other heritable traits (32). However, other plant studies of highly heritable traits contain missing heritability (66, 149). Much of this missing heritability is thought to stem from underpowered statistical associations, small allele effect sizes, and the need for stringent significance cutoffs to control type I errors (74), which has led to the notion that only a portion of heritability can be explained via the SNPs used in a GWA study (i.e., SNP heritability) (159). Exemplifying this, even with ~5.4 million individuals saturating SNP heritability, half of human height variation remained unexplained (163). Quantitative genetic studies have led to the omnigenic model of inheritance (74), where there are a few large-effect genes that either directly influence the trait or serve as network master regulators (Figure 2d). These genes are in turn regulated by peripheral genes, which themselves integrate into the organismal gene regulatory network, such that much phenotypic variation is explained by variants with infinitesimally small network effects that remain unidentified. Although GWA studies have provided many insights into the control of trait inheritance, alternative techniques need to be developed to discover these peripheral regulators and their genetic interactions in order for models to explain all trait heritability.

## BULK MOLECULAR PROFILING OBSCURES THE CELLULAR CONTEXT OF GENETIC VARIANTS

The proliferation of GWA studies has identified innumerable causative loci implicated in myriad phenotypes. However, defining the precise molecular and cellular mechanisms that translate GWA loci to phenotypic variation remains challenging. In humans, up to 95% of disease risk variants have been attributed to noncoding sequences (151), implicating transcriptional regulation as the driver of phenotypic diversity. Indeed, recent studies show that eQTL and chromatin accessibility QTL (caQTL) overlap considerably with disease-associated variants (38, 43). Similarly, plant bulk-tissue QTL studies for molecular phenotypes including DNA methylation (30, 57, 117) and transcription abundance (62) significantly coincide with organismal phenotype–associated variants. Bulk-tissue population-scale genetic investigations of molecular traits lack the resolution to pinpoint the cellular context in which a given genetic variant has an effect, particularly for variants affecting narrow developmental windows or rare cells (145). Single-cell population genomic association can circumvent these shortcomings, providing unprecedented insight into where and when genetic variants contribute to phenotypic variation (Figure 2e). However, single-cell genomic association studies are underpowered (due to data sparsity) compared to their bulk counterparts with similar individual numbers (145).

## ANIMAL SINGLE-CELL MOLECULAR QUANTITATIVE TRAIT LOCUS STUDIES HAVE REVEALED THE IMPACTS OF CELL TYPE-RESTRICTED VARIANTS

Single-cell genomic association methods have been developed in animal models to identify the cellular and developmental contexts of disease-associated variants. For example, a study of human endoderm differentiation from 125 individuals leveraged scRNA-seq to identify developmentally or cellularly restricted eQTL for 1,833 genes (~17% of genes tested) (21). The importance of development and cell contexts has been repeatedly corroborated for eQTL (60, 91, 100, 161), and caQTL appear to be influenced similarly (7). Highlighting the relevance of cell type–restricted variants to continuous phenotypes, another single-cell genomic association found 95 developmental and cellular context–specific eQTL near established disease-associated loci, including a known basal cell carcinoma risk factor (13). Similarly, studying midbrain development from 215 donors across three time points identified an overlap of context-specific eQTL with 1,284 disease-associated variants (54). Indeed, the relevance of context-specific eQTL to disease has only been revealed through single-cell population genomics (142). Through single-cell plant genomic associations, it will be critical to determine whether genetic variants underlying agronomic trait variation are also restricted to narrow developmental and cellular contexts.

## APPLICATIONS OF PLANT SINGLE-CELL TRANSCRIPTOME AND CHROMATIN ACCESSIBILITY PROFILING

To date, single-cell genomic associations remain unimplemented in plants. However, commercial scRNA-seq products have facilitated many plant scRNA-seq studies. These scRNA-seq experiments, particularly of *Arabidopsis* roots (25, 53, 113, 119, 122, 154, 167), established proof of principle, paving the way for plant single-cell genomics and revealing developmental transcriptomic patterns. Supporting the translational value of these root atlases, scRNA-seq of rice roots found strong conservation in the developmental programs between rice and *Arabidopsis* roots, particularly for cells with meristematic identity (166). Plant scRNA-seq has moved beyond atlas production to address pointed biological questions. For example, transcriptomic data from over 110,000 cells from wild type and 2 cell identity mutants, *shortroot (shr)* and *scarecrow (scr)*, revealed putative *trans*-differentiation of cortical to endodermal cell identity in *scr* and aberrant vascular cell identities in *shr* backgrounds (119). Likewise, analysis of *Arabidopsis* seed snRNA-seq established strikingly dynamic and heterogeneous parental imprinting with distinct spatial and cell cycle–dependent patterning (102). scRNA-seq approaches have been applied to important crops, including maize (68, 92, 93, 115, 134, 158) and rice (73, 166). scRNA-seq of maize shoot apices revealed mechanisms that contribute to the genomic integrity of plant stem cells and defined the transcriptional program driving early shoot differentiation (115). Two maize scRNA-seq studies followed meiotic development to reveal the transcriptional changes coordinating cell-state transitions and the timing of the sporophyte-to-gametophyte transcriptional transition (92, 93). scRNA-seq of developing maize inflorescence revealed the proximity between cell identity genes and yield GWA loci (158), hinting that variation in agronomic traits could indeed be biased toward restricted developmental and cellular contexts such as human disease (142). Construction of more single-cell atlases across the plant kingdom will define the conserved and derived transcriptional programs underlying the differentiation of plant cells.

In contrast to scRNA-seq, plant scATAC-seq chromatin accessibility profiling is still in its infancy, having only been implemented in *Arabidopsis* roots (29, 34, 141) and a maize organ panel (81). These initial studies have resulted in several discoveries. For example, scATAC-seq of *Arabidopsis* roots revealed positive correlations between chromatin accessibility and nuclear gene transcription predictive of cell identity (34). Independent integration of *Arabidopsis* root scATAC-seq and scRNA-seq identified which expressed transcription factors putatively controlled chromatin accessibility at specific loci (29). Exhibiting the power of combined scRNA- and scATAC-seq, this study also discovered three endodermal subtypes with distinct cell identities and predicted functions, which were undetected by scRNA-seq alone (29). In maize, cell type–specific ACRs, especially in floral tissues, had both signs of selection and increased association with phenotype-associated GWA variants (81). These findings suggest that, as in animals, variation within cell type–restricted genes and ACRs may disproportionately drive phenotypic diversity. Future studies combining scATAC- and scRNA-seq will undoubtedly lead to appreciable discoveries in plants. Importantly, tandem scRNA- and scATAC-seq genomic association approaches will reveal more about the molecular mechanisms and spatiotemporal cellular context in which extant genetic variation controls phenotype manifestation.

## FUTURE APPLICATION OF SINGLE-CELL PLANT POPULATION GENOMICS

Single-cell plant genomic association offers several advantages over conventional bulk-tissue approaches: (*a*) Single-cell platforms profile cell states without requiring transgenic or endogenous cell-type marker genes, which do not exist for most plant species. (*b*) Cell-type proportions in single-cell data reflect endogenous in planta cell-type distributions (however, biased nuclei/cell recovery can be a major issue; see 134) that can serve as a secondary phenotype, allowing cell-type frequency comparisons between genotypes. (*c*) Single-cell eQTL and caQTL mapping enables the dissection of genetic variant effects in diverse cellular states (e.g., cell type, developmental or cell cycle stage, and ploidy), enabling QTL identification in rare cell states that is not possible through bulk approaches. Single-cell genomic associations will be more powerful when utilizing scRNA- and scATAC-seq multiomics. Multiomic measurements of large, genetically diverse cohorts will disentangle the relationships among genetic variants, chromatin landscapes, transcription dynamics, cell types, and quantitative traits. It seems likely that some plant cell types will have a disproportionate influence on specific phenotypes, mirroring animal models. Discovering these cell-to-phenotype interactions will further our understanding of plant phenotypic control and provide novel targets for biotechnological or breeding manipulation to improve crop traits. Beyond finding future breeding targets, comparing wild and domesticated subpopulations will provide insights into how millennia of human selection have altered plant genomes, potentially revealing pan-species domestication targets.

## SOMACLONAL VARIATION CAN FUEL SINGLE-CELL GENETIC ASSOCIATION WITH MOLECULAR TRAITS

Instead of relying on individual-level genetic diversity, single-cell genetic associations can examine somaclonal variation to determine how molecular phenotypes become varied in genetically divergent cell lineages. Somaclonal variation arises from the accumulation of mutations in distinct somatic lineages within an individual, leading to functional differences within cells of the same identity. In animals, somaclonal variation is linked to physiological aging and disease (36, 139). Single-cell genetics has already been applied to ascertain how mammalian genome sequences drift somatically (139, 157). Multiomics will enable the measurement of these somatic mutations and their effects on molecular phenotypes such as chromatin accessibility and gene expression, better revealing how specific somaclonal changes contribute to aging and disease. Investigation into mammalian somatic variants has developed tools to detect polymorphisms from single-cell data (89) or build de novo references at the cellular level (157), which can be translated to plant systems.

Plants have a rich research history involving somaclonal variation, and several components intrinsic to plant biology leave it well-poised to combine single-cell genetics with somaclonal variation. Plant somaclonal variation was first described in the early twentieth century (9), and somaclonal variation was later used to map out the patterns of cell divisions during development (87, 104, 114, 127). Flowering plant meristems are divided into two or three layers; the outermost layer, the L1, gives rise to all epidermal tissue, while the inner L2 layer gives rise to most organs, including most gametes (84). Meristematic cell divisions are arranged such that the L1, L2, and sometimes L3 (114) layers maintain distinct cellular lineages, except for layer invasion events (132), during somatic development. In vegetatively propagated plants, the accumulation of mutations in each layer produces stable chimeras. Some crops, notably grape, have been vegetatively propagated for centuries or longer, leading to functional clonal diversity that has been selected upon for modern grape improvement (146). Perhaps the most extreme example of clonal variation is *Vitis vinifera* cv. Pinot Noir, which may predate the Roman conquest of Gaul (~50 BC) and has many clonal variants, most with chimeric L1/L2 layers (48). These L1/L2 sectors represent the culmination of hundreds of years of somatic changes and selection from one ancestral genome. Single-cell genetics provides the utility to separate the signals from these chimeric clones, determining how these mutations affect chromatin accessibility or gene expression and providing a foundational model to study the process, and ramifications, of the genesis of novel somatic variation.

## QUANTITATIVE GENETICS IS RESTRICTED BY STANDING VARIATION

The results of any population genetics approach are intrinsically restricted by the genetic variation present in the study population; one can only evaluate the impact of the alleles captured in a chosen population, and quantitative genetic studies using a distinct population will find different genetic interactions. Different QTL studies examining the same phenotype in different populations often find different trait-associated loci. For example, study of human height found different QTL (44, 67, 152), although studies that use a large and diverse enough population can discover most common variants underlying extant trait variation (163). Likewise, studying giant Gough Island mice revealed size-controlling loci that were not found in other populations (41). Large-effect alleles can be subpopulation restricted, which can occur due to interspecific introgression, as in the cases of a hypoxia tolerance allele introgressed from Denisovans into Tibetans (50) and altered timing of the floral transition by an inverted haploblock introgressed between *Helianthus* spp. (sunflower) (140). Population fixation of important alleles can also obfuscate important trait regulators. For example, cultivated maize contains a *Teosinte Branched1 (Tb1)* variant fixed during domestication, and only a recombinant population derived from its wild ancestor, teosinte, revealed how *Tb1* controls branching (27). Alternatively, fixing a large-effect QTL can allow for the discovery of other smaller-effect loci (155). Mirroring these studies, the evaluation of diverse populations via single-cell genomic associations will reveal more of the genetics behind complex traits.

Although population selection can ameliorate the issue, single-cell genomic association on standing variation limits what can be learned about the genetics controlling any phenotype, as extant variants only affect a small fraction of the true genetic architecture that manifests complex traits. The standing variation in a wild or domesticated population only encompasses polymorphisms that survive natural or artificial selection, respectively; this often excludes variants in key trait regulators, precluding their discovery through mapping or association. To illustrate this, we return to the GWA of maize flowering (10). Buckler et al. (10), despite explaining 89% of maize time-to-flowering heritability, found no variants associated with *Indeterminate1 (Id1)*, a potent regulator of flowering (10, 19, 123). Loss-of-function *id1* alleles flower extremely late, which is thought to produce a reproductive barrier, preventing variants from existing in wild or domesticated maize. Instead, the role of *Id1* in maize flowering was only uncovered through mutational approaches (19, 123). Using mutagens to create populations was critical in mapping many physiologically important genes, including genes involved in the floral transition such as *LEAFY* (153) and those involved in specifying essential cell types such as *MUTE* (103), that were intolerant of mutation outside of a lab. Mirroring the value of mutations in classical genetics, we anticipate that the merger of single-cell mutant populations with single-cell genomic measurement will be a powerful technique to interrogate the genetic control of molecular traits, notably transcript levels, independent of extant population variation. We believe these single-cell genetics approaches will transform our understanding of what controls molecular phenotypes throughout the genome.

## TRANSITIONING FROM SINGLE-CELL GENOMICS TO SINGLE-CELL GENETIC SCREENS WILL FURTHER INTERROGATE THE GENETIC ARCHITECTURE OF COMPLEX TRAITS

Single-cell genetics is being applied to mammalian systems, through techniques such as Perturb-seq (26). Perturb-seq utilizes a pool of barcoded CRISPR guide RNA sequences that are introduced randomly to individual cells containing CAS9 to disrupt endogenous gene expression, followed by scRNA-seq to determine both the CRISPR target sequence(s) and the effects of the cassette on the transcriptome (26). This high-throughput approach, when using dCAS9, allows the individual repression [CRISPR interference (CRISPRi)] of every expressed gene in a cell type (111), and variants have been developed based on ectopic mRNA expression (143). Perturb-seq, which requires cytoplasmic RNA to deconvolute the guide RNA sequences post hoc, is difficult to adapt to plant systems due to the challenges and technical artefacts of isolating and manipulating protoplasts. In particular, it remains challenging to limit the number of guide RNA sequences introduced to each protoplast. As such, alternative single-cell genetic methods must be developed and applied to plant systems.

**Figure 3:**
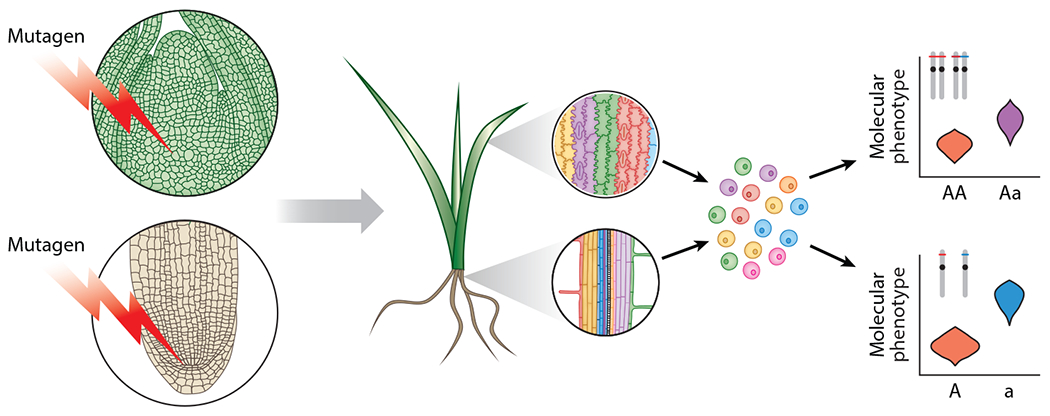
Use of single-cell mutant populations to generate and map functional genetic diversity throughout the genome. Mutagenesis provides an opportunity to interrogate more of the genetic architecture underpinning a trait than exists in extant populations. Mutagenesis of meristems creates chimeric cells, each with a unique set of mutations that impact molecular phenotypes such as gene expression and chromatin accessibility. Subsequent development will create chimeric sectors that share a set of mutations (*rainbow colors*). Multiomic measurement of these sectors will measure both molecular phenotype and mutations, the latter of which can be merged from repeated measurements of cells from the same sector to build sector genotypes. Since the chances of inducing the exact same mutation are poor, a rare-variant collapsing strategy can be used to pool sectors with similar mutations, bolstering minor allele frequencies. Then, phenotypic comparison between unaltered (A; *red*) and mutant (a; *blue*) alleles can reveal the loci that influence the measured molecular trait. Although it may be possible to find and associate heterozygous mutations in diploids (*top*), haploidy (*bottom*) will greatly simplify this scheme, simplifying mutation detection and improving mutation penetrance. A similar scheme can be applied to cell lines in nonplant systems.

The difficulties in adapting high-throughput CRISPR-CAS9 screens to plants lead us to propose the use of more traditional mutagens, such as chemical mutagens, or ionizing radiation to generate genetically diverse mutant cell populations to associate loci with molecular phenotypes, such as mRNA transcription, via single-cell genetic screens. However, to associate individual mutations with changes in transcription, the induced mutations must be detectable at the cellular level concurrent with transcriptome measurements. Multiomic scRNA- and scATAC-seq have great potential here, with scATAC-seq measuring induced mutations over most of the functional genome, and scRNA-seq measuring the subsequent transcriptional changes. However, the high sparsity of these multiomic measurements remains an impediment to mutation detection when screening mutant cell populations. To address this sparsity problem, mutagenized cell populations can be measured after cell proliferation. For example, when exposed to multicellular plants, mutagens create chimeras, with each mutagenized cell containing independent mutations (Figure 3). From this chimera, mutant meristematic cells divide to produce clonal sectors with unique genotypes, and sampling these sectors aids mutation detection by allowing the amalgamation of repeated measurements of cells or nuclei from the same genotype. Additionally, since plants share metabolites through their symplasm, chimeric sectors carrying mutations in some core metabolism genes should be recoverable, and studiable, even though they would be lethal if fixed organism-wide. A complementary approach to improve mutation detection is to mutagenize haploid cells; in haploids, only one allele is present, facilitating cell genotype determination with fewer reads. Another benefit of mutagenizing haploids is that all mutations become penetrant, easing the study of mutant alleles. Although common in plants but not in animals, the isolation and maintenance of haploid mammalian cell lines and individuals have increased in prevalence (33, 69), allowing similarly designed single-cell mutant population screens in animals.

Induced mutation creates alleles that have large, often very deleterious, effects and allow the discovery of regulatory relationships between loci that are invariant in organism-level populations due to fitness constraints. Notably, this large allele effect size should empower the detection of *trans* regulators that, presumably due to the large phenotypic consequences of their alteration, often contain only small-effect variants within species (147). Although mutant alleles will often have large effects, they will also have an exceptionally small minor allele frequency (MAF) in the cellular population, with the chances of inducing the exact same mutation being near zero for large genomes. This low MAF will hamper the ability to find significant associations between mutants and transcriptional variants. Large-effect, but rare, alleles are a common occurrence in natural populations (62, 105), and allele pooling strategies that treat variants in similar genomic features (e.g., in the same exon or gene) as comparable alleles have been successful in mapping human disease-associated variants (105). In a similar way, pooling mutant alleles within the same exons, genes, or ACRs will bolster MAF, increasing the power of single-cell mutant population screens to identify associations with expression variation.

Single-cell mutant genetic screens will confer distinct advantages compared to the quantitative genetic study of domesticated or wild individuals: (*a*) Induced mutation will generate large effect-size alleles in important genomic loci that would be too maladaptive to exist in individuals. (*b*) Large cell populations can reside in Petri dishes or chimeric individuals, conferring significant space, and cost, savings compared to equivalent numbers of individuals. (*c*) Mutant tolerance will vary by cell type (these survivorship approaches are elaborated upon below), revealing the specific cellular contexts in which CREs and genes become indispensable. (*d*) As all mutations will be independent, there will be no underlying population structure between alleles, deconvoluting linked genetic effects, including those in low recombination regions. (*e*) Cell types should tolerate mutations that are lethal due to developmental (e.g., embryonic) defects, revealing pleiotropic roles of genes unstudiable in their fixed forms. (*f*) Detection of causal mutations, and not polymorphisms linked to a functional variant, will ease the process of linking genes or CREs to phenotypes. These advantages position single-cell mutant screens to reveal much about the *cis* and *trans* transcriptional regulators functioning in different cell types. This approach will synergize with established single-locus and classical population genetics techniques, further unveiling the intricate relationships behind omnigenic trait heritability.

Since single-cell mutant screens involve the de novo generation of genetic diversity, they will open new horizons to expand quantitative genetics techniques to organisms with poor genetic diversity. For example, domesticated animals, such as cattle, contain runs of homozygosity (101) where past selection and inbreeding have eliminated genetic diversity in large, linked regions. The strong historic selection on these runs of homozygosity highlights their agronomic importance, yet the lack of diversity greatly impedes examining any underlying function. Similarly, wild populations that have recovered from near extinction events have dramatically reduced extant genetic diversity (42, 96). Here, the creation of single-cell mutant populations circumvents the need for standing variation and furthers the use of quantitative genetics to study the genetic control of traits in these recalcitrant systems.

Single-cell mutant screens will also help to overcome long generation times, which remain common obstacles in individual-level quantitative genetics techniques. Perennial crops are plagued by this problem, with seed-sown (recombinant) individuals often taking years to reach sexual maturity. Additionally, many perennial cultivars share extensive coancestry (88, 90, 150), limiting genetic diversity within elite germplasm. These problems impede quantitative genetic approaches, slowing both the basic understanding of perennial plant genetics and the process of breeding their improvement. Again, single-cell mutant populations will bypass these limitations, improving our understanding of perennial plant genetic architecture.

Single-cell genetics will enable novel experimental approaches that were previously infeasible. For example, in mutant cell populations, especially haploid ones, the integral parts of the genome will be revealed by low mutation recovery rates. This genomic survivorship analysis will reveal the many genomic regions that are integral to all cell viability. Additionally, a cell type–specific survivorship analysis will reveal the loci required to generate or maintain different cell fates. This single-cell mutant survivorship analysis can be combined with abiotic or biotic stresses to highlight the genes, CREs, and cell types needed to endure these conditions.

Single-cell genomics also provides a high-throughput framework to exploit *Arabidopsis* T-DNA insertional mutant libraries (2, 97), which exist for most genes. Cells from many plants with mapped T-DNA insertion lines can be extracted en masse and measured via single-cell genomics. By treating cells as individuals, and devising a T-DNA line pooling and detection strategy, single-cell measurement of expression and/or chromatin accessibility in these established T-DNA may address the dearth of experimentally validated functions of plant genes (112). Beyond *Arabidopsis*, mutant libraries have been created for other plant models such as maize (83, 86), allowing for the translation of this scheme to diverse species. These are just a few examples outlining how single-cell genetics provides an opportunity for creative and new high-throughput experimental approaches to address a wide range of outstanding biological questions.

## CHALLENGES AND TECHNOLOGICAL ADVANCES FACING SINGLE-CELL TECHNOLOGY

Single-cell genomics is currently expensive, making many studies cost prohibitive. Therefore, reducing costs is a major priority that will expand its user base and promote discovery. This situation is analogous to the emergence of high-throughput sequencing, where technological advances rapidly increased the number of reads produced per run; Illumina sequencing output changed from ~30 million 32-bp reads to 25 billion paired-end 150-bp reads today. This improvement significantly reduced costs and thereby democratized its research use. Similar efforts are underway to reduce single-cell genomics costs by expanding the number of cells or nuclei that are obtained per experiment. The biggest gains in cell numbers have resulted from combinatorial indexing or preindexing samples and overloading microfluidic instruments. Droplet single-cell combinational ATAC-seq (dsci-ATAC-seq) (65) and single-cell combinatorial fluidic indexing (scifi)-RNA-seq (23) both achieved >100,000 cells per library, increasing cell output 10- to 25-fold. Other approaches to increasing cell throughput have utilized additional rounds of barcoding, including sci-ATAC-seq3 (28) and sci-RNA-seq3 (12). These advances enable deeper coverage of samples and/or using multiple samples per library, depending on the pre-indexing steps. Although these methods are designed to measure chromatin accessibility and mRNAs, future innovations in library preparation will enable measurement of additional genomic features such as histone modifications, chromatin interactions, transcription factor binding, and other molecular traits.

The process of annotating cell types, which requires characterized cell type–specific markers, remains challenging, especially in poorly studied organisms. Spatial-omics provides one solution to improving cell-type annotations. For example, 10× Genomics’ Visium Spatial Gene Expression images, sections, and sequences mRNA while fixing the mRNA coordinates in the tissue. This identifies transcripts in their spatial context, increasing the accuracy of cell-type annotation, both on the slide and in any accompanying single-cell data. Beyond the restrictions imposed by prohibitively high costs, current 10× spatial resolutions, ~55 μm, are limiting as they are larger than most plant cells. However, ~500-nm resolution transcriptomics measurements have been achieved through spatial enhanced resolution omics-sequencing (Stereo-seq) (14, 156). Another tool, plant hybridization-based targeted observation of gene expression map (PHYTOMap) (95), provides an alternative spatial method to measure cell type–specific mRNAs. This method uses multiplexed fluorescent probes for in situ hybridization, identifying cell type–specific expression of target genes in a three-dimensional context. PHYTOMap is currently limited to a few target genes, but its labeling scheme can expand to thousands. Vizgen offers another high-resolution in situ hybridization technique, but currently it only assays ~500 predesignated transcripts. The ability to produce cell type–specific measurements from >100,000 cells per library combined with emerging spatial-omics will lead to high-confidence cell-type atlases, enabling accurate and rapid cell-type annotation in subsequent experiments.

## FUTURE APPLICATIONS OF EMERGING SINGLE-CELL TECHNOLOGIES

Single-cell genomics is in its infancy, and the number of cells or nuclei profiled from each experiment continues to rise. We see routine outputs changing from ~10^4^ cells to at least 10^5–6^ cells per run in the coming years. This increased throughput will allow approaches analogous to those done on bacterial cells in culture, impacting the types of questions that can be addressed and future experimental design. Most studies currently measure limited developmental stages and genotypes due to cost and limiting cell/nuclei yield. However, future studies will examine plant processes over developmental progression, in response to many environments and treatments and across genetically distinct individuals. In particular, eQTL and caQTL studies from biparental populations or diversity panels will identify the cell types in which genetic variants act to alter important continuous phenotypes such as yield and stress response. This unprecedented resolution will reveal much about how genotype shapes complex phenotypes.

There is a natural tendency to replace assays such as bulk RNA-seq with scRNA-seq within pre-existing experimental designs, which will certainly lead to greater resolution, especially for signals from rare cell types. However, the transition from single-cell genomics to single-cell genetics offers opportunities to break the experimental mold by turning cells into individual test tubes, allowing truly unprecedented experiments. As described earlier, mutagenesis can be used to study gene and regulatory element function at cell-type resolution. Individual cells can also be altered via chemical or environmental treatments or permuted ectopic gene expression (143), such as transcription factors, and then assayed via single-cell genomics. The advent of single-cell genetics will identify which cell types elicit what molecular responses to these many stimuli, accelerating our understanding of how gene regulatory networks in different cells integrate to produce phenotypes. Although it is a future endeavor, it is important to recognize the potential for single-cell genetics to further our understanding of cell type–specific processes, as we expand our toolkit to push the boundaries of biological discovery.

## Abbreviations

Heritability: the proportion of a population’s phenotypic variance that can be explained by genetics
Single-cell genetics: the combination of cellular-level genetic manipulation with single-cell phenotypic measurements
Chromatin accessibility: the measure of DNA unbound by nucleosomes or other DNA-binding proteins
Multiomic: measurement of two or more omic metrics from the same nucleus or cell
Data sparsity: data with a high proportion of missing information (dropouts) per measurement
Protoplast: a plant cell isolated from its cell wall
Plasticity: the ability of one genotype to produce diverse phenotypes in response to the environment
Genetic assimilation: the process through which a previously environmentally induced response becomes constitutive
Ploidy: the number of chromosomes sets in a cell
Endoreduplication: DNA duplication without subsequent cell division
Introgression: the process in which a haplotype is moved from one species or subpopulation into another through hybridization followed by recurrent backcrosses
Type I errors: rejecting the null hypothesis when it is really true (false positives in statistical tests)
Population structure: the coinheritance of groups of alleles due to shared ancestry and genetic linkage
Chimera: an individual that contains two or more distinct genotypes
Symplasm: the shared cytoplasm of multiple plant cells that are connected by plasmodesmata pores
T-DNA: the piece of DNA integrated into plant genomes by *Agrobacterium tumefaciens*
Spatial-omics: an omics measurement integrated with information about position within an organism
